# Arsenic sulfide as a potential anti-cancer drug

**DOI:** 10.3892/mmr.2014.2838

**Published:** 2014-11-03

**Authors:** WENPING DING, LIAN ZHANG, SUNGKYOUNG KIM, WEI TIAN, YINGYING TONG, JIANWEN LIU, YONG MA, SIYU CHEN

**Affiliations:** 1 Department of Oncology, Xinhua Hospital Affiliated to Shanghai Jiao Tong University School of Medicine, Shanghai 200092, P.R. China; 2 State Key Laboratory of Bioreactor Engineering and Shanghai Key Laboratory of New Drug Design, School of Pharmacy, East China University of Science and Technology, Shanghai 200237, P.R. China; 3 Department of Dermatology, Shanghai Third People’s Hospital, School of Medicine, Shanghai Jiao Tong University, Shanghai 201999, P.R. China

**Keywords:** arsenic sulfide, anti-cancer activity, solid tumor

## Abstract

Arsenic sulfide (As_4_S_4_) is the main component of realgar, which is widely used in traditional Chinese medicine. Previous studies have shown the beneficial effects of As_4_S_4_ in the treatment of hematological malignant diseases, however, its effects on solid tumors have yet to be fully elucidated. The current study aimed to explore the anti-cancer effect and the mechanism of As_4_S_4_ on solid tumors *in vitro* and *in vivo*. Cells from four human solid tumor cell lines, including the MKN45 gastric cancer cell line, the A375 malignant melanoma cell line, the 8898 pancreatic carcinoma cell line and the HepG2 hepatocellular carcinoma cell line, were treated with As_4_S_4_ *in vitro*, using the L02 embryonic liver cells as a control. The efficacy of As_4_S_4_ was assessed *in vivo* using mice implanted with Lewis lung carcinoma cells. The results of the current study demonstrated that As_4_S_4_ significantly inhibited the proliferation of solid tumor cells in a dose- and time-dependent manner, but produced a less pronounced effect on L02 cells. Additionally, As_4_S_4_ was observed to induce apoptosis (including morphological changes and an enhanced sub-G_1_ population), which was accompanied by the activation of caspase-3 and −9. Furthermore, treatment with As_4_S_4_ significantly inhibited the growth of implanted tumors in mice. These results suggest that As_4_S_4_ possesses potent *in vitro* and *in vivo* antitumor activity via the induction of cell apoptosis.

## Introduction

As a traditional Chinese medicine, arsenic has been widely used for over 2,000 years. It is effectively used in traditional remedies for the treatment of inflammation, ulcers, convulsions and schistosomiasis, and studies have demonstrated that arsenic produces positive effects in cancer therapy (1–3). One study demonstrated that arsenic trioxide (As_2_O_3_) was clinically effective in patients with acute promyelocytic leukemia (APL) (4). As_2_O_3_ was approved for the therapy of APL in the year 2000, and subsequently has been widely used therapeutically in liver, cervical and esophageal solid tumors (5–7). A number of studies have demonstrated that the induction of apoptosis and inhibition of proliferation are involved in the antitumor mechanism of As_2_O_3_ in hematopoietic malignancies and solid tumors (8–11).

Arsenic sulfide (As_4_S_4_), another arsenic compound, is the main active component of realgar, an orange-red crystalline mineral that has been extensively used in traditional Chinese medicine. Compared with As_2_O_3,_ As_4_S_4_ is less toxic, but may elicit a similar anti-neoplastic action. The therapeutic potential of arsenic sulfide in malignancies, particularly hematopoietic tumors, has been the focus of a number of studies (12–15). Its antitumor effects are associated with the inhibition of proliferation, apoptosis and the suppression of BCR-ABL oncoprotein activity (16,17). However, the action of As_4_S_4_ as a treatment for solid tumor is unclear. Thus, the aim of the current study was to investigate the role of As_4_S_4_ in the treatment of solid tumors and its potential as an anticancer agent. In the present study, the anti-cancer effects of As_4_S_4_ were investigated *in vitro* and *in vivo*.

## Materials and methods

### Materials

A total of 6 g As_4_S_4_ was dissolved in 200 ml RPMI 1640 medium (Gibco Life Technologies, Carlsbad, CA, USA) for 12 h, then the concentration of arsenic was measured by atomic absorption spectrometry (IRIS 1000, Thermo, Waltham, MA, USA). The 3-(4,5-dimethylthiazol-2-yl)-2,5-diphenyltetrazolium bromide (MTT) was purchased from Sigma-Aldrich (St. Louis, MO, USA).

### Cells and animals

The MKN45 gastric cancer, A375 malignant melanoma, 8898 pancreatic carcinoma, HepG2 hepatocellular carcinoma and L02 embryonic liver cell lines were purchased from the cell bank of the Type Culture Collection of The Chinese Academy of Sciences (Shanghai, China). All cells were cultured in RPMI 1640 medium supplemented with 10% heat-inactivated fetal bovine serum (FBS; Gibco Life Technologies), penicillin (100 U/ml) and streptomycin (100 U/ml; both Gibco Life Technologies). Cells were incubated at 37°C in a humidified atmosphere of 95% air and 5% CO_2_. Male C57BL/6 mice (n=32; age, six weeks), were obtained from the Animal Center of Fudan University (Shanghai, China; license no., 2007-0002 SCXK). Mice implanted with Lewis lung carcinoma (LLC) cells were purchased from the Institute of Materia Medica, Chinese Academy of Sciences (Shanghai, China; license no., SCXK 2004-0002). Mice were maintained in an animal facility under pathogen-free conditions (license no., SYXK 2003-0031). This study was approved by the ethics committee of Xin Hua Hospital Affiliated to Shanghai Jiao Tong University School of Medicine (Shanghai, China).

### Cytotoxicity assay

The cytotoxicity assay was performed using MTT. Cells were seeded into and allowed to attach to 96-well culture plates (1×10^4^ cells/well), for 4 h prior to treatment. As_4_S_4_ at concentrations of 0, 1.25, 2.5, 5 and 10 *μ*g/ml was administered to the cells. After 24-h treatment, cell viability was evaluated by MTT assay. MTT solution (20 μl; Sigma-Aldrich) was added to each well and the plates were incubated for an additional 4 h at 37°C. The supernatant from each well was then carefully removed, and 100 μl dimethyl sulfoxide (Sigma-Aldrich) was added to each well and thoroughly mixed. The optical density (OD) was measured on a Model 550 microplate reader (Bio-Rad Laboratories, Hercules, CA, USA) at an absorbance wavelength of 492 nm and a reference wavelength of 630 nm. It was denoted that the percentage of cell viability = (average OD of experimental group/average OD of control group) × 100%. The experiment was repeated three times. The IC_50_ (concentration causing 50% inhibition of cell growth compared with the control) value of As_4_S_4_ for each of the tumor cell lines was also calculated after 24 h.

### Determination of time-activity curve

The effect of As_4_S_4_ on cell viability was determined by measuring the MTT absorbance of living cells, which were seeded in and allowed to attach to 96-well plates. Following 0, 6, 12, 24, 36 and 48 h incubation of the tumor cells with As_4_S_4_ (at IC_50_) while L02 cells with 10 μg/ml As_4_S_4_, the cell viability was evaluated by MTT assay. The experiment was repeated three times.

### Hematoxylin and eosin (HE) staining assay

Cells from exponentially growing cultures were seeded in 24-well culture plates and treated with As_4_S_4_ (at IC_50_) for 36 h, and L02 cells were treated with 10 *μ*g/ml As_4_S_4_. Cells were washed with phosphate-buffered saline (PBS; Gibco Life Technologies), fixed in 4% paraformaldehyde (Sigma-Aldrich) for 15 min, stained with hematoxylin (Beyotime Institute of Biotechnology, Jiangsu, China) for 8 min, washed again with PBS, stained with eosin (Beyotime Institute of Biotechnology) for 5 min, and then examined and imaged with with the Nikon Eclipse 55i microscope (Nikon Corporation, Tokyo, Japan).

### Flow cytometric analysis of cellular DNA content

Three cell lines (A375, MKN45 and L02) were seeded in 6-well culture plates (2×10^5^ cells/well). Following 12-h incubation, A375 and MKN45 cells were treated with the respective IC_50_ of As_4_S_4_ for 36 h, and L02 cells were treated with 10 *μ*g/ml As_4_S_4_. Floating and attached cells were collected in centrifuge tubes. Cells were washed with PBS, then resuspended and fixed in 70% ice-cold ethanol (Yangyuan, Changshu, China) for 4 h at 4°C. Subsequently, they were treated with RNase A (50 *μ*g/ml; Sigma-Aldrich) for 30 min. Cells were stained with propidium iodide (50 *μ*g/ml), then analyzed in a flow cytometer (BD Accuri C6, BD Biosciences, Franklin Lakes, NJ, USA). The percentages of cells in the G_0_/G_1_, S, G_2_/M and sub-G_1_ phases were analyzed using standard ModiFit LT 3.1and CellQuest Pro software (BD, Mac OS X.1; San Jose, CA, USA).

### Lactate dehydrogenase (LDH) release assay

The A375 and MKN45 cells were treated with the respective IC_50_ doses of As_4_S_4_, and L02 cells were treated with 10 μg/ml As_4_S_4_. After 36 h, supernatants were harvested to measure the levels of LDH using the LDH kit (BHKT Clinical Reagent Co., Beijing, China)

### Caspase activity assay

MKN45 cells were seeded in 10-cm dishes. Following a resting period of 12 h, cells were treated with various concentrations (0 μg/ml, 0.25 × IC_50_, 0.5 × IC_50_, 1 × IC_50_) of As_4_S_4_ for 36 h. Following treatment, the cells (floating and attached) were collected and washed three times with PBS and resuspended in 50 mM Tris-HCl (pH 7.4; Sigma-Aldrich), 1 mM EDTA (Sigma-Aldrich) and 10 mM ethyleneglycoltetraacetic acid (Sigma-Aldrich). Cell lysates were clarified by centrifugation at 18,000 × g for 3 min and clear lysates containing 50 *μ*g protein were incubated with 100 *μ*M enzyme-specific colorigenic substrates (Ac-DEVD-pNA; Beyotime Institute of Biotechnology) at 37°C for 1 h. The activity of caspase-3 and −9 was denoted as the cleavage of colorimetric substrate measured at an absorbance of 405 nm.

### In vivo experiments with C57BL/6 mice

The mice implanted with LLC cells were sacrificed by cervical dislocation. Under sterile conditions, tumor tissues were dissected and the tumor cells were suspended in RPMI 1640 medium containing 10% FBS. The cell suspension was injected into the flanks of the experimental mice (10^6^ cells in 200 μl PBS for each mouse). The tumor-bearing mice were divided into four groups: Negative control (NC) group, positive control group and high- and low- dose groups, each containing eight mice. The tumor-bearing mice were administered with an intraperitoneal injection of either 30 (low) or 60 (high) mg/kg dose of As_4_S_4_, daily for eight days. The NC group was treated with 0.9% normal saline (Rongbai, Shanghai, China) and the positive group was treated with 20 mg/kg cyclophosphamide (CTX; Yili, Shanghai, China), respectively. Subsequent to euthanization, the solid tumors were harvested and weighed, and blood was drawn to measure the level of interleukin-2 (IL-2). The solid tumor weights were statistically analyzed. The rate of inhibition (RI) was calculated according to the following formula: RI = [(mean tumor weight of the NC group - mean tumor weight of the drug group)/mean tumor weight of the NC group] × 100%.

### Statistical analysis

Each experimental value was expressed as the mean ± standard deviation. Statistical analysis was performed using Origin software, version 7.0 (OriginLab, Northampton, MA, USA) to evaluate the differences between groups. P<0.05 was considered to indicate a statistically significant difference.

## Results

### Cytotoxic effect of As_4_S_4_ on tumor cells

**Figure 1:**
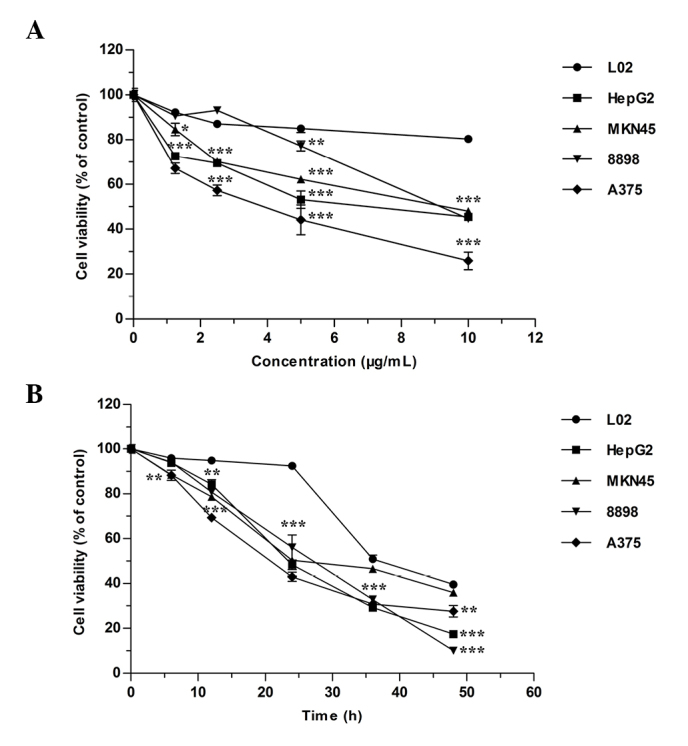
Cytotoxic effect of As_4_S_4_ on 8898, A375, HepG2 and MKN45 cancer cells and L02 healthy liver cells. (A) Viability of the cells was measured by MTT assay following exposure to As_4_S_4_ at different concentrations (1.25–10 *μ*g/ml) for 24 h. (B) Measurement of cell viability following treatment with the respective IC_50_s of As_4_S_4_ for each tumor cell line for different time periods (0–48 h) (L02 cells were treated with 10 μg/ml As_4_S_4_). Data are expressed as the mean ± standard deviation. The cell proliferation was inhibited by As_4_S_4_ in an (A) dose-dependent and (B) time-dependent manner (P<0.001) and each cell line presented a different sensitivity to the inhibitory effect of As_4_S_4_ (^*^P<0.05, ^**^P<0.01 and ^***^P<0.001, vs. L02 cells). As_4_S_4_, arsenic sulfide.

The cytotoxicity assay results for As_4_S_4_ on the five cell lines are presented in Fig. 1. The data indicated that the cell proliferation was inhibited by As_4_S_4_ in a dose-dependent (Fig. 1A) and time-dependent (Fig. 1B) manner (P<0.001), and each cell line presented a different sensitivity to the inhibitory effect of As_4_S_4_. The IC_50_ values of the tumor cell lines following 24-h treatment are presented in Table I. As_4_S_4_ generated a weaker effect on L02 cells compared with the four tumor cell lines.

### Effect of As_4_S_4_ on cell morphology

**Figure 2:**
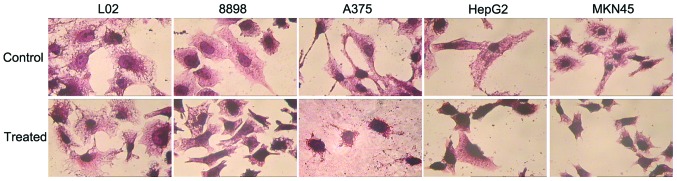
Hematoxylin and eosin staining of 8898, A375, HepG2 and MKN45 cells cultured in the presence or absence of the respective IC_50_s of As_4_S_4_ for 36 h and L02 cells treated with 10 *μ*g/ml As_4_S_4_. Condensed and fragmented nuclei were observed in the As_4_S_4_-treated tumor cells, but not in the L02 cells. Magnification, ×400. As_4_S_4_, arsenic sulfide.

The HE staining assay identified that the tumor cells (8898, A375, HepG2 and MKN45) treated with As_4_S_4_ exhibited cell shrinkage, nuclear condensation and fragmentation, which are typical characteristics of apoptosis. However, the treated L02 cells did not exhibit significant morphological changes (Fig. 2).

### Effect of As_4_S_4_ on G_2_/M phase arrest and the apoptotic sub-G_1_ population

**Figure 3:**
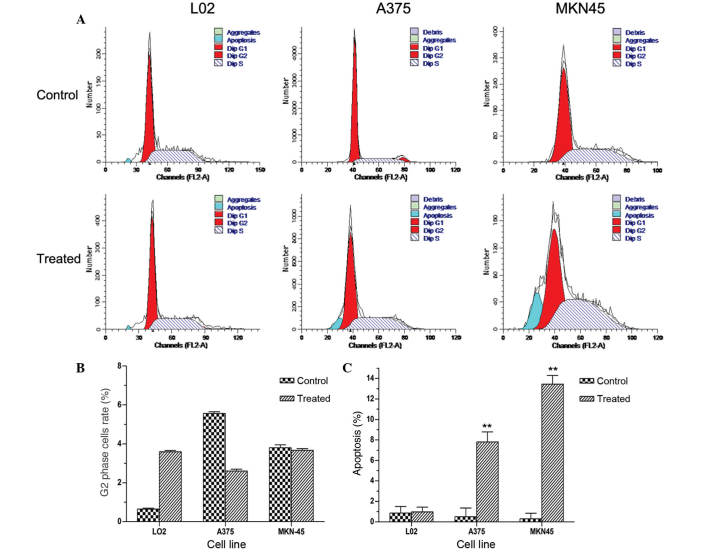
Effect of As_4_S_4_ on G_2_/M phase and apoptotic cells. (A) L02, A375 and MKN45 cells were treated with or without As_4_S_4_ and analyzed by flow cytometry. (B) Data indicated no clear G_2_/M phase arrest in the tumor or L02 cells treated with As_4_S_4_. (C) A significant increase in apoptotic sub G1 population was observed in As_4_S_4_-treated A375 and MKN45 cells, but not in the L02 cells. Data are expressed as the mean ± standard deviation; ^*^P<0.05, ^**^P<0.01 and ^***^P<0.001 vs. control. As_4_S_4_, arsenic sulfide.

To determine whether the reduction in cell viability observed involved alterations to the cell cycle, the effect of As_4_S_4_ on the cell cycle distribution in the A375, MKN45 and L02 cell lines was investigated using fluorescence-activated cell sorting analysis (Fig. 3A). The apoptotic index was calculated by measuring the number of cells in the sub-G_1_ population following treatment with As_4_S_4_. Subsequent to exposure of A375, MKN45 cells to the respective IC_50_s of As_4_S_4_ and of L02 cells to 10 μg/ml As_4_S_4_ for 36 h, no marked G_2_/M phase arrest was observed, as demonstrated in Fig. 3B. These results suggest that As_4_S_4_ produced no significant effect on G_2_/M phase arrest. A significant increase in the sub-G_1_ fraction was identified in tumor cells treated with As_4_S_4_, whilst no significant difference was observed in the L02 cells compared with the untreated control cells (Fig. 3C).

### Effect of As_4_S_4_ on the level of LDH and the activation of caspase

**Figure 4:**
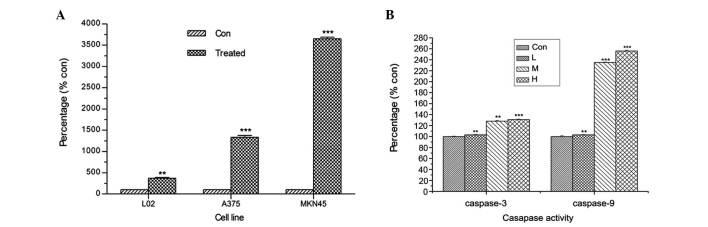
Effect of As_4_S_4_ on the release of LDH and the activation of caspase-3 and −9. (A) Subsequent to exposure of A375 and MKN45 cells to the respective IC_50_s of As_4_S_4_ for 36 h, and exposure of L02 cells to 10 *μ*g/ml As_4_S_4_, the leakage of LDH from cells was analyzed. (B) Caspase-3 and −9 activation was analyzed in MKN45 cells treated with different concentrations of As_4_S_4_ for 36 h: Control, 0 μg/ml; L, 0.25 × IC_50_; M, 0.5 × IC_50_ and H, 1 × IC_50_. Data are expressed as the mean ± standard deviation; ^*^P<0.05, ^**^P<0.01 and ^***^P<0.001 vs. control. As_4_S_4_, arsenic sulfide; LDH, lactate dehydrogenase; L, low; M, medium; H, high.

The LDH release assay measured the leakage of LDH into the extracellular medium following cellular lysis. The release of intracellular LDH was detected following exposure to As_4_S_4_ (Fig. 4A). Meanwhile, caspase-3 and −9 were activated in MKN45 cells treated with As_4_S_4_ for 36 h (Fig. 4B). These results indicate the involvement of caspase-3 and −9 in As_4_S_4_-mediated cell apoptosis.

### As_4_S_4_ inhibits the growth of solid tumors and elevates the levels of IL-2 in blood

**Figure 5:**
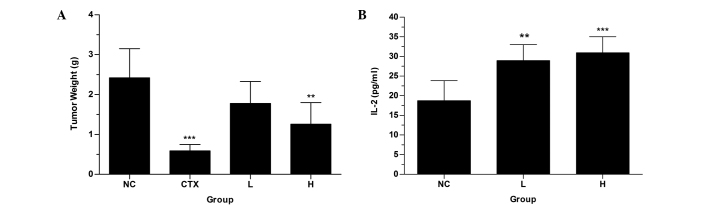
As_4_S_4_ inhibited tumor growth and elevated the levels of IL-2 in the tumor-bearing C57BL/6 mice. (A) Tumor weight reduced significantly following treatment with 60 mg/kg (H) of As_4_S_4_, but the reduction observed was not significant with 30 mg/kg (L). (B) Levels of IL-2 were significantly elevated in the treatment groups compared with the NC group. Data are expressed as the mean ± standard deviation; ^*^P<0.05, ^**^P<0.01 and ^***^P<0.001 vs. NC. As_4_S_4_, arsenic sulfide; IL-2, interleukin 2; L, low; H, high; NC, negative control; CTX, cyclophosphamide.

Mouse lung cancer LLC cells were implanted in C57BL/6 mice. Following treatment with As_4_S_4_ for eight days, the suppression of tumor growth was observed (Fig. 5A). The inhibition ratios of the low-dose group (As_4_S_4_, 30 mg/kg) and high-dose group (As_4_S_4_, 60 mg/kg) were 26.45 and 47.93%, respectively (Table II). The high dosage exhibited a greater anticancer effect than the low dosage, and produced a significantly reduced tumor weight compared with that of the NC group. In addition, As_4_S_4_ treatment did not significantly alter the body weight of the mice (data not shown). Subsequent to treatment with As_4_S_4_, the concentrations of IL-2 in the treatment groups were higher compared with the NC group (Fig. 5B). These results demonstrate that As_4_S_4_ is able to suppress tumor growth.

## Discussion

As_4_S_4_ has attracted worldwide interest in recent years due to the successful clinical application of arsenic compounds in the treatment of APL (12,18). However, its efficacy in the treatment of solid tumors remains to be thoroughly elucidated. Hence, in the present study, the antitumor effect of As_4_S_4_ on solid tumors and its possible mechanism of action were investigated.

Apoptosis, the most common form of tumor cell death, is a biological process of programmed cell death (PCD). The typical morphological and molecular changes that occur during the course of apoptosis include cell shrinkage, nuclear fragmentation, chromatin condensation, DNA fragmentation and changes in apoptotic protein expression (19–21). In the current study, using MTT assay, As_4_S_4_ was observed to be able to inhibit the proliferation of tumor cells in a dose- and time-dependent manner, but produced a less marked effect on healthy L02 cells, which were used as a control to assess the hepatotoxicity of As_4_S_4_. Flow cytometry indicated that the inhibition of A375 and MKN45 tumor cell growth by As_4_S_4_ may be due to cell apoptosis. However, no significant effect of As_4_S_4_ on G_2_/M phase arrest was observed. The HE staining assay demonstrated that four tumor cell lines treated with the IC_50_ of As_4_S_4_ for 36 h exhibited cell shrinkage, nuclear condensation and fragmentation (typical characteristics of apoptosis), while L02 cells treated with As_4_S_4_ demonstrated no differences prior to and post administration.

LDH is clinically significant as a marker of injury and disease, since it is released during cell or tissue damage. The LDH release assay measures the leakage of the soluble cytoplasmic LDH enzyme into the extracellular medium via cellular lysis. The current study demonstrated that As_4_S_4_ increased the release of LDH, indicating the induction of apoptosis or necrosis.

Caspases are cysteine proteases activated by a cascade, involving the cleavage of their precursors. Caspase-3 is an executioner caspase that disassembles cells through the cleavage of proteins such as PARP, in order to inactivate them. Caspase-3 is commonly activated by either the caspase-9-mediated mitochondrial pathway, or by the caspase-8-mediated death receptor pathway (22–24). In the current study, As_4_S_4_ treatment was demonstrated to lead to caspase-3 and −9 activation, which suggests the involvement of the caspase pathways in As_4_S_4_-induced apoptosis.

The antitumor activity of As_4_S_4_ was further examined in a mouse model. Similar to the *in vitro* results, As_4_S_4_ was capable of inhibiting tumor growth *in vivo*. In the high dosage group, the tumors grew slower compared to the NC group. However, As_4_S_4_ did not produce a significant inhibitory effect on tumor growth in the low As_4_S_4_ dosage group compared with the NC group.

Studies have demonstrated that IL-2 serves an important function in the regulation of antigen-specific T-cell responses (25,26). The cytokines expressed by a T-cell in response to an antigen indicate the specific pathway, and IL-2 is associated with the T helper 1 (Th1) pathway. IL-2 has been demonstrated to serve an important role in specific immunological responses to tumor cell growth (27,28). Thus, in the present study, IL-2 production was investigated to evaluate the hypothesis that As_4_S_4_ increases T-cell activation by modulating IL-2. The results demonstrated that the serum concentrations IL-2 were enhanced in As_4_S_4_-treated C57BL/6 mice bearing LLC, compared with the NC mice, suggesting that it may be a potent inducer of Th1-type cytokines.

In the present study, As_4_S_4_ was observed to induce apoptosis in cancer cells. The detailed mechanism of apoptosis induction in solid tumors by As_4_S_4_ requires further investigation. Previous studies have reported that As_4_S_4_ treatment induced differentiation of hematological tumor cells (29,30), suggesting that the antitumor action of As_4_S_4_ in solid tumors may involve cellular differentiation.

In the current study, the results suggested that As_4_S_4_ may be able to inhibit cell growth and increase the release of LDH. Furthermore, the apoptosis was suggested to involve caspase-3 and −9 activation. Additionally, As_4_S_4_ was demonstrated to have the effect of suppressing tumor growth *in vivo*. These results suggest that As_4_S_4_ may be a potential therapeutic candidate in the treatment of solid tumors.

## Figures and Tables

**Table I:** IC_50_ value of arsenic sulfide for each tumor cell line following 24-h treatment.

Cell line	IC_50_ (*μ*g/ml) × ± standard deviation
HepG2	6.89±1.078
MKN45	9.37±0.948
8898	9.06±0.984
A375	3.78±0.827

**Table II:** Antitumor effect of As_4_S_4_ *in vivo*.

Group	Injection dosage (mg/kg) × ± standard deviation	Tumor weight (g)	Tumor inhibition (%)
Negative control	0	2.42±0.73	0
Positive control (cyclophosphamide)	20	0.59±0.16^b^	75.62
High As_4_S_4_	60	1.26±0.54^a^	47.93
Low As_4_S_4_	30	1.78±0.55	26.45

C57BL/6 mice were inoculated with Lewis lung carcinoma cells (10^6^ cells/mouse), injected intraperitoneally with As_4_S_4_ for eight days and subsequently sacrificed. Data are expressed as the mean ± standard deviation.

a P<0.01;

b P<0.001 vs. negative control.

As_4_S_4_, arsenic sulfide.
